# Shipworm bioerosion of lithic substrates in a freshwater setting, Abatan River, Philippines: Ichnologic, paleoenvironmental and biogeomorphical implications

**DOI:** 10.1371/journal.pone.0224551

**Published:** 2019-10-31

**Authors:** J. Reuben Shipway, Gary Rosenberg, Gisela P. Concepcion, Margo G. Haygood, Charles Savrda, Daniel L. Distel

**Affiliations:** 1 School of Biological Sciences, University of Portsmouth, Portsmouth, United Kingdom; 2 Ocean Genome Legacy Center, Department of Marine and Environmental Science, Northeastern University, Nahant, MA, United States of America; 3 Academy of Natural Sciences, Drexel University, Philadelphia, PA, United States of America; 4 Marine Science Institute, University of the Philippines, Diliman, Quezon City, Philippines; 5 Department of Medicinal Chemistry, University of Utah, Salt Lake City, UT, United States of America; 6 Department of Geosciences, Auburn University, Auburn, AL, United States of America; Universidade do Vale do Taquari - Univates, BRAZIL

## Abstract

Teredinid bivalves, commonly referred to as shipworms, are known for their propensity to inhabit, bioerode, and digest woody substrates across a range of brackish and fully marine settings. Shipworm body fossils and/or their borings, which are most allied with the ichnotaxon *Teredolites longissimus*, are found in wood preserved in sedimentary sequences ranging in age from Early Cretaceous to Recent and traditionally they have been regarded as evidence of marginal marine or marine depositional environments. Recent studies associated with the Philippine Mollusk Symbiont International Collaboration Biodiversity Group (PMS-ICBG) expedition on the island of Bohol, Philippines, have identified a new shipworm taxon (*Lithoredo abatanica*) that is responsible for macrobioerosion of a moderately indurated Neogene foraminiferal packstone cropping out along a freshwater reach of the Abatan River. In the process of drilling into and ingesting the limestone, these shipworms produce elongate borings that expand in diameter very gradually toward distal termini, exhibit sinuous or highly contorted axes and circular transverse outlines, and are lined along most of their length by a calcite tube. Given their strong resemblance to *T*. *longissimus* produced in wood but their unusual occurrence in a lithic substrate, these shipworm borings can be regarded as incipient *Gastrochaenolites* or, alternatively, as *Apectoichnus*. The alternate names reflect that the borings provide a testbed for ideas of the appropriateness of substrate as an ichnotaxobasis. The discovery of previously unrecognized shipworm borings in lithic substrates and the co-occurrence of another shipworm (*Nausitora*) in submerged logs in the same freshwater setting have implications for interpreting depositional conditions based on fossil teredinids or their ichnofossils. Of equal significance, the Abatan River study demonstrates that macrobioerosion in freshwater systems may be just as important as it is in marine systems with regard to habitat creation and landscape development. *L*. *abatanica* serve as ecosystems engineers in the sense that networks of their abandoned borings provide habitats for a variety of nestling invertebrates, and associated bioerosion undoubtedly enhances rates of mechanical and chemical degradation, thus influencing the Abatan River profile.

## Introduction

Teredinid bivalves are colloquially referred to as shipworms and, more informally, as the “termites of the sea” [1]. These monikers reflect the elongate, vermiform body common to teredinids and the well-known propensity of these bivalves to bioerode and digest various lignocellulosic substrates—natural woody materials (driftwood, rhizomes, seeds) and manufactured wooden structures (ships, piers, docks)—in brackish to fully marine settings [1]. The presence of shipworms in the stratigraphic record, which for teredinids extends from Jurassic time through to the present [1–2], is commonly manifested by occurrences of fossil wood containing their elongate, axially contorted, acutely clavate borings that, in most cases, have been assigned to the ichnospecies *Teredolites longissimus* [3–4]. The presence of *Teredolites*-bored fossil wood in stratigraphic packages traditionally has been taken as evidence for deposition in marginal marine or marine paleoenvironments [5–10].

Recent studies demonstrate that teredinids are not strictly limited to life in woody substrates [11] or to marine or brackish settings. New work completed as part of the Philippine Mollusk Symbiont International Collaboration Biodiversity Group (PMS-ICBG) expedition on the island of Bohol, Philippines, has led to the description and naming of a novel shipworm taxon that is responsible for extensive macrobioerosion of a *lithic* substrate (limestone) cropping out in the bed and banks beneath *freshwaters* of the Abatan River. This new taxon is described in detail elsewhere [12]. The objectives of this contribution are to: (1) describe the character of the substrates and the borings therein; (2) compare these shipworm borings with previously defined ichnotaxa commonly ascribed to bivalves; (3) discuss the implications of this new finding for paleoenvironmental inferences traditionally drawn from fossil occurrences of shipworms and/or their biogenic structures; and (4) address the potential roles of shipworms as ecosystem engineers and geomorphic agents in freshwater aquatic systems.

### Geologic setting

**Fig 1:**
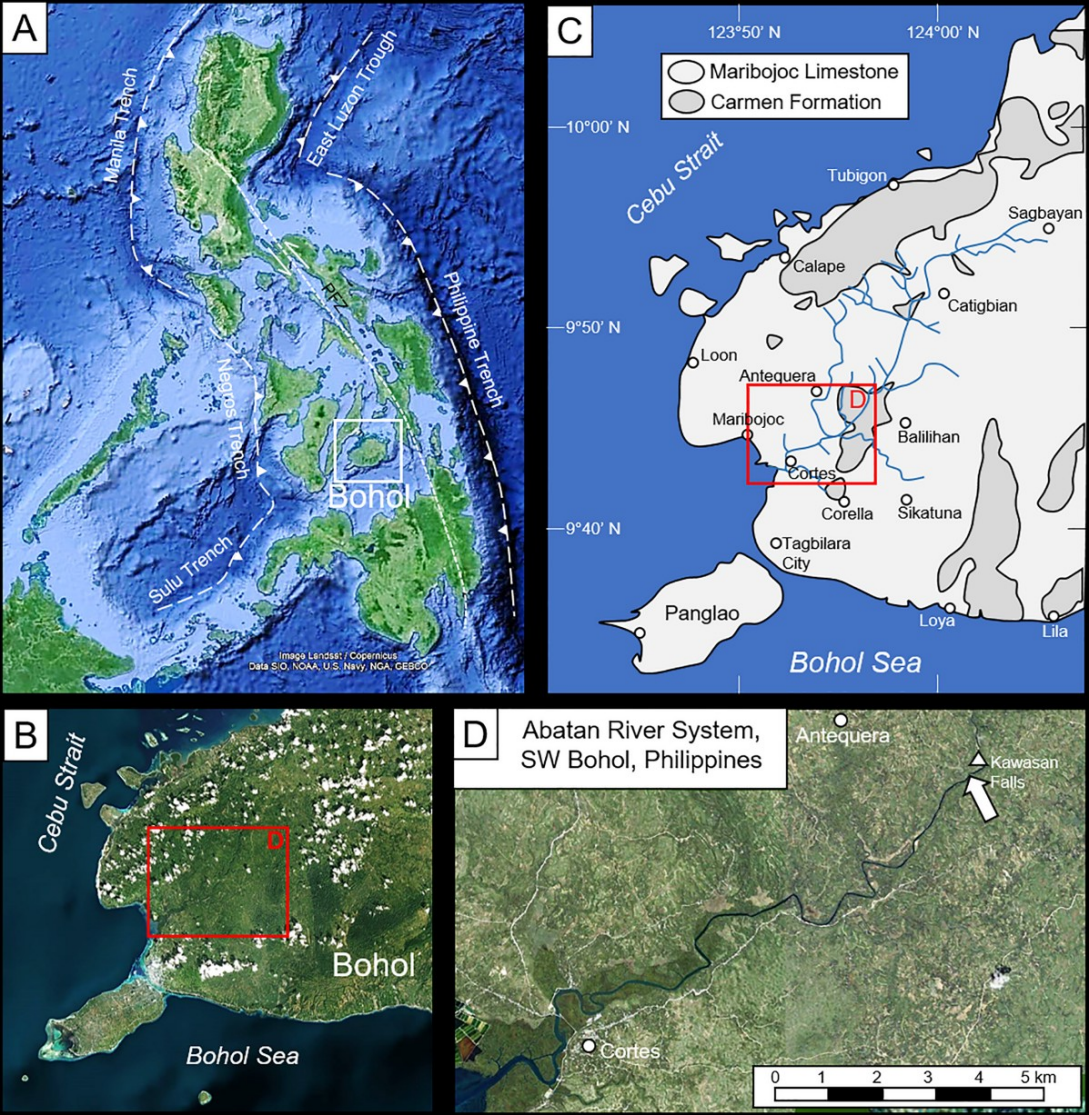
Location of study. (A) The Philippine archipelago showing position of Bohol Island (white box) PFZ = Philippine Fault Zone. (B) Southwest Bohol Island. (C) Generalized geologic map of southwestern Bohol showing distribution of Miocene Carmen Formation and Plio-Pleistocene Marijoboc Limestone (modified from PPDOBOHOL). Black box in B-C shows position of study area shown in D. (D) The Abatan River system and study site (arrow) below Kawasan Falls (coordinates 9°45’58.3”N, 123°56’40.9”E). Satellite images used in A-B, D were produced using https://www.nauticalcharts.noaa.gov/ENCOnline/enconline.html.

Bohol is a central Visayan island in the south-central part of the Philippine mobile belt (Fig 1A and 1B). The island includes Mesozoic basement rocks locally overlain by Miocene and younger sediments and minor volcanics (e.g., [13–14]). Basement rocks comprise the Alicia Schist, Cansiwang melange of magmatic rocks and deep marine sediments, and Southeast Bohol Ophiolite complex, all of purported Cretaceous age. Over a large part of the island, basement rocks are variably overlain by the Lower to Middle Miocene Carmen Formation, which includes the Anda Limestone, Pansol Clastic, and Lumbog Volcaniclastic members, locally intruded by the Jagna Andesite; Upper Miocene-Lower Pliocene Sierra Bullones Limestone; and/or Upper Pliocene to Pleistocene Maribojoc Limestone.

In the current study area in southwestern Bohol, the surface geology is dominated by relatively undeformed carbonates of the Anda and Maribojoc limestones (Fig 1C), the erosion of which forms the spectacular conical karst features (i.e., mogotes) of Bohol’s Chocolate Hills. Both of these units locally crop out in the ~350 km^2^ drainage basin of the Abatan River, which meanders west-southwestward to its estuarine mouth at Cortes (Fig 1D). The bioeroded substrates described herein are exposed along the Abatan River just below Kawasan Falls, 14.8 km (river distance) upstream from the coast (Fig 1D). Detailed descriptions of stratigraphy in the immediate area are not available. However, based on the geologic map produced by the Bohol Provincial Planning and Development Office (https://ppdo.bohol.gov.ph/maps/thematic-maps/geologic-map/) and brief petrologic descriptions provided by Faustino et al. [14], these substrates are provisionally assigned to the Anda Limestone Member of the Miocene Carmen Formation.

### Location and description of bioeroded substrates

**Fig 2:**
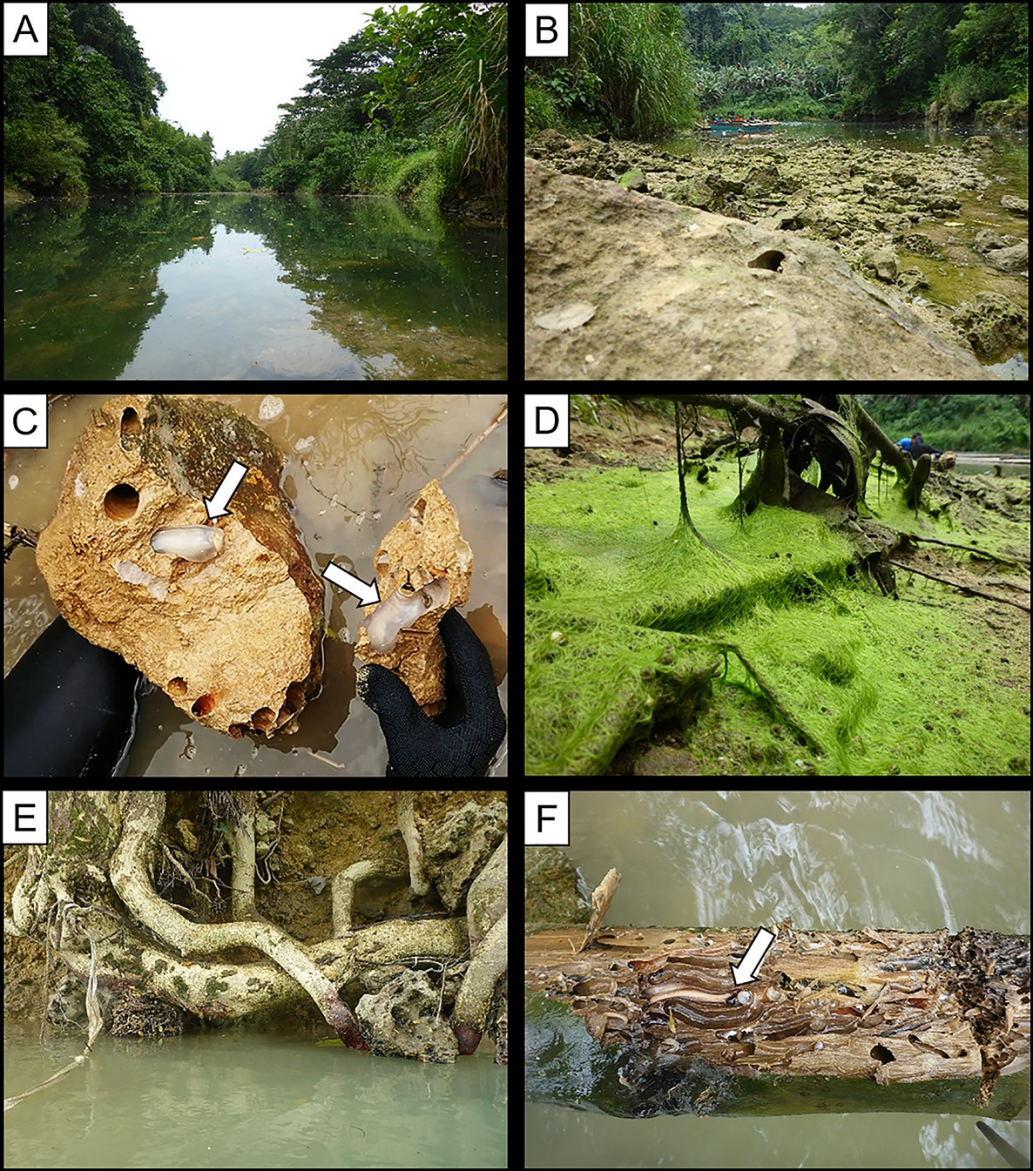
Photographs of the study site. (A) Abatan River bordered by heavily vegetated floodplain. (B) Exposed limestone bedrock and associated rubble. (C) Stone-boring shipworm *Lithoredo abatanica* in limestone bedrock. (D) Limestone outcrops variably coated by alga. (E) Exposed tree roots along banks of Abatan River. (F) Abundant wood clasts on submerged stream bed colonized by wood-boring shipworm *Nausitora* sp.

The limestone substrates addressed in this study are exposed on the bed and low banks of a short (~20 m long) and narrow stretch of the Abatan River, bounded on both sides by vegetated floodplain (Fig 2A and 2B). River widths and depths at this locality vary with seasonal precipitation and tidal stage. At the time of field observations (August 17–19, 2018), which were made just prior to the rainy season, maximum river depth was ~5 m, tidal range was ~50 cm, river widths varied from 5–10 m along the studied stretch, and waters were fresh; measured salinities remained <0.5 ppt through tidal cycles. Limestone outcrops on the river bed and low banks are heavily colonized (Fig 2C) by the shipworm *Lithoredo abatanica* [12] and variably coated with algae and strewn with loose bioeroded limestone cobbles and boulders (Fig 2B and 2D). Woody land plant roots (Fig 2E) and submerged logs (Fig 2F) are heavily colonized by the wood-boring shipworm *Nausitora* sp. (Fig 2F).

**Fig 3:**
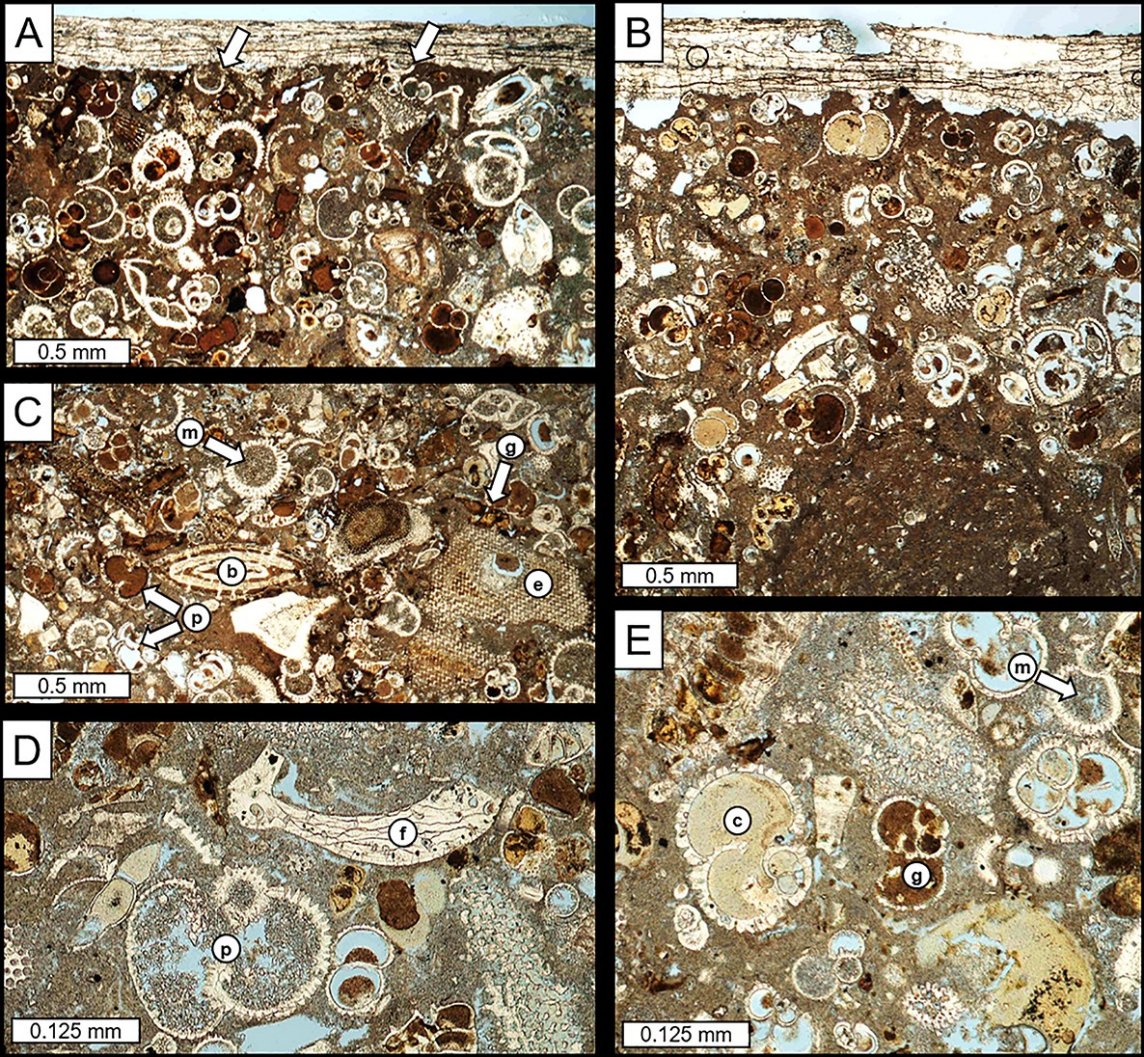
Photomicrographs of limestone substrate. (A, B) packed biomicrite and associated *L*. *abatanica* tube linings (top of each photo). Arrows in A highlight thin-shelled foram tests that have been truncated at the boring wall. Darker pod of sparse biomicrite in lower part of B is a burrow fill or intraclast. (C) Planktonic (p) and benthonic (b) forams and echinoderm fragments (e). Intraparticle pores are filled with micrite (m) and brown Fe-rich cement (goethite?; g). (D) Planktonic foram (p) and microbored bivalve fragment (f). (E) Open intraparticle pores (blue) and intraparticle pore-filling cements, including micrite (m), goethite? (g), and chamosite? (c).

The bored substrate is a moderately indurated, yellowish gray limestone. As shown in representative photomicrographs (Fig 3), the rock is a packed biomicrite (foraminiferal packstone) with localized, small (1–2 mm), roughly ovate pods of finer-grained sparse biomicrite (foraminiferal wackestone) that represent either intraclasts or burrow fills (Fig 3B). Skeletal allochems, ranging from 0.1 to 1.0 mm in size and surrounded by fine micritic matrix, are dominated by a variety of planktonic and benthic foraminifera. Other common yet subordinate skeletal components include echinoderm, gastropod, and sponge-bored bivalve fragments (e.g., Fig 3). Macroporosity is mainly limited to primary intraparticle pores (e.g., foram chambers, echinoderm stereomic pores; Fig 3D) but much of this porosity has been occluded by cements. Although micritic and microsparitic calcite fills are observed locally, pore-filling cements are dominated by Fe-rich minerals (goethite and, possibly, chamosite) (Fig 3E), which also locally infiltrate the micrite matrix.

Rock textures and composition indicate that this limestone accumulated in a clastic-free marine setting. The predominance of sand-sized skeletal allochems suggests that the depositional environment was influenced, at least periodically, by bottom currents. The unusual Fe-rich pore-filling cements likely precipitated in response to redox reactions with Fe- and organic carbon-rich river and/or ground waters after uplift of the limestone.

### The shipworms

**Fig 4:**
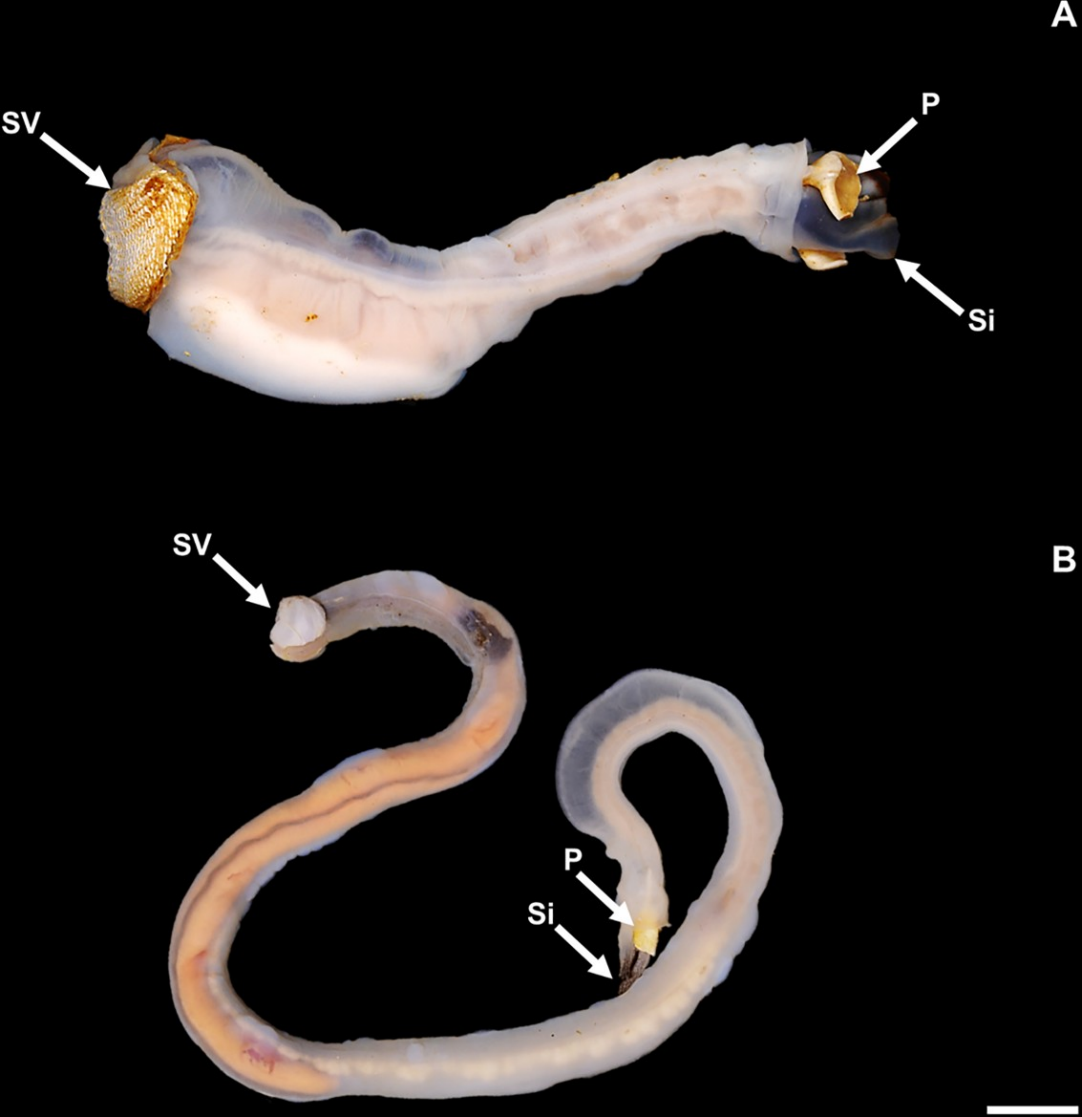
Shipworms from the Abatan River. (A) Specimen of stone-boring shipworm *Lithoredo abatanica* extracted from limestone. (B) Specimen of wood-boring-shipworm *Nausitora* sp. extracted from submerged wood clast. P = pallets; Si, siphons; SV, shell valves. Scale bar = 1 cm.

Limestone macroborings at the Abatan River site are all attributed to shipworms, which on the basis of hard part morphology, soft tissue anatomy, and molecular phylogenetic data represent a recently described genus and species—*Lithoredo abatanica* [12]. Like most teredinids, this new form is characterized by a long vermiform body, small valves at the anterior end, calcite tube linings, and siphons equipped with a pair of calcite pallets that allow the tight sealing of tunnel entrances (Fig 4).

Among the 69 specimens of *L*. *abatanica* extracted from their tunnels and measured in the field, contracted body lengths and widths range from 5.5 to 105.4 mm and 4 to 35 mm, respectively. However, based on the diameters of empty borings observed on loose or excavated limestone blocks, even larger individuals likely inhabit the substrate, particularly beneath the subtidal river bed, which could not be easily sampled.

In addition to the rock-boring shipworm *L*. *abatanica*, all submerged woody substrates, including logs, palms and tree roots, were colonized by the wood-boring shipworm *Nausitora* sp. (Fig 4B). Populations of *Nausitora* sp. extended from the freshwater collection site of the rock-boring shipworms, into brackish waters further downstream.

Part of this work was completed under the supervision of the Department of Agriculture Bureau of Fisheries and Aquatic Resources, Philippines (DA-BFAR), in compliance with all required legal instruments and regulatory issuances covering the conduct of the research. All Philippine specimens used in this study were obtained using Gratuitous Permit 0140–17 issued by DA-BFAR

### The borings

**Fig 5:**
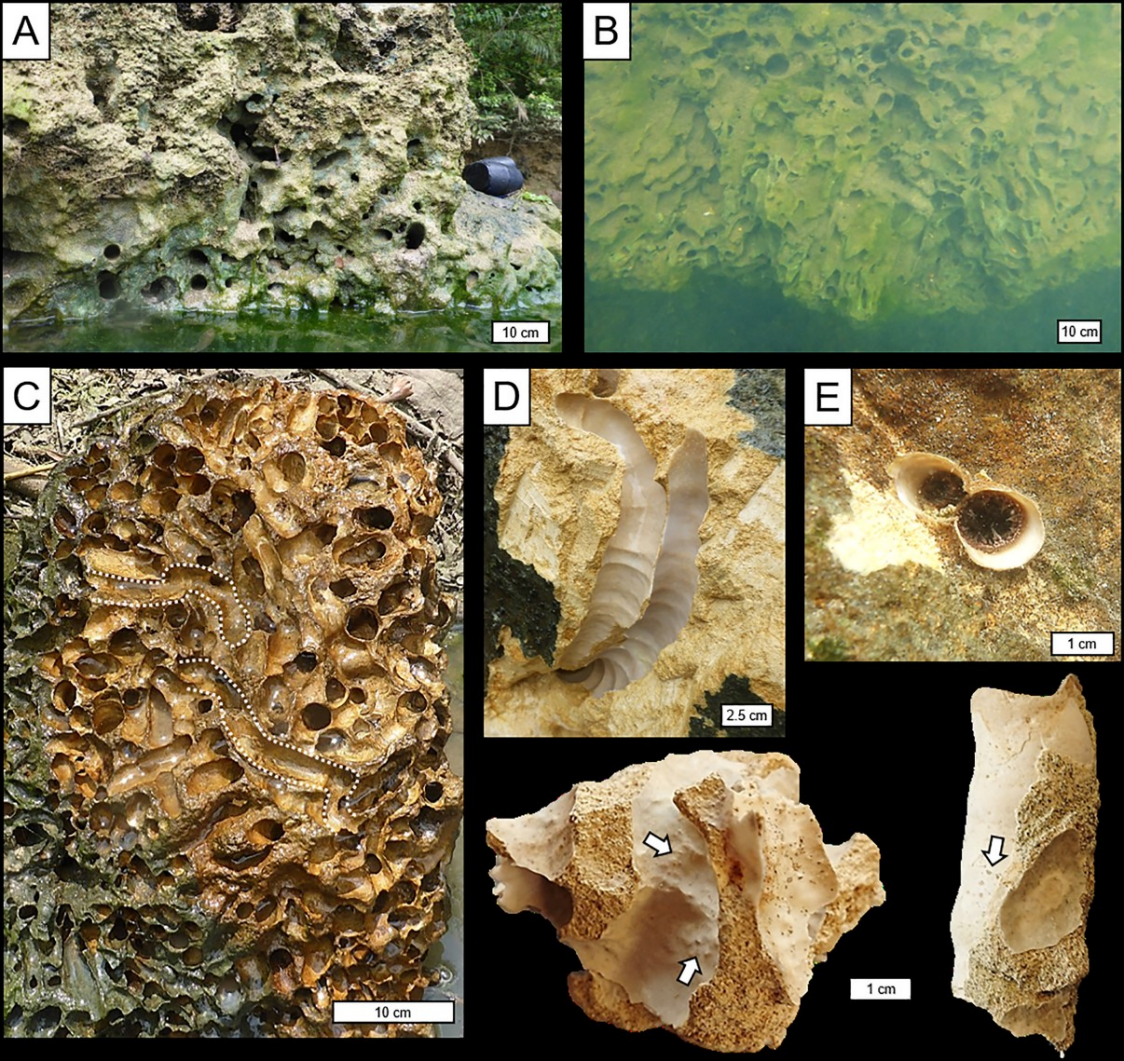
Borings of *Lithoredo abatanica*. (A, B) Borings exposed in river bank at low tide (A) and on submerged algae-coated limestone outcrop (B). (C) Densely emplaced borings (now vacated) in a limestone clast. Note tortuous paths of many borings (e.g., those partly outlined by white dashed lines). (D) Closely-spaced, calcite lined borings. Note axial distortion of the tunnel and transverse annulations in calcite lining. (E) Calcite-lined tunnel aperture showing figure-eight configuration, through which *Lithoredo abatanica* siphons protrude. (F, G) Close-up images of calcite tube interiors (F) and exterior (G) along with remnant limestone host rock (scale = 1 cm). Tiny xenoglyph bumps and pits, indicated by arrows in F and G respectively, reflect precipitation of the tube over larger sand-sized grains in the substrate.

Field observations of borings were made on *in situ* limestone substrates exposed along the river bank at low tide (Fig 5A), on *in situ* blocks excavated from the subtidal river bed (Fig 5B), and on loose cobble- and boulder-sized clasts eroded from the river bank and bed (Fig 5C). Supplementary notes on the character of borings were made on relatively small samples collected for subsequent laboratory analyses.

All examined substrates were consolidated limestone and were moderately to extensively riddled with *L*. *abatanica* borings (Fig 5A–5C). Where borings are densely emplaced, the tracemakers do not penetrate older borings or active borings of their nearest neighbors. However, individual tunnels are commonly separated only by a very thin (≤1 mm) partition of remnant substrate (Fig 5C), and in some cases, substrate is altogether absent between tunnels; i.e., the calcite tube linings (described below) of adjacent borings are in direct contact (Fig 5D).

High densities of borings make it difficult to fully characterize individual tunnels in their entirety. Nonetheless, surfaces exposed by natural erosion or manual splitting of substrate blocks allowed for general characterization of boring morphology. Borings were generally initiated normal to exposed substrates surfaces, which vary from near vertical in river banks to horizontal on the river bed. Axes of established borings are highly variable; some are nearly straight or gently curved, but many are meandering or tortuous (Fig 5C and 5D), in part reflecting phobotaxis and competition for limited substrate space.

Although views of complete specimens were rarely availed on examined surfaces, axial lengths of partially exposed tunnels of mature *L*. *abatanica* commonly exceed 30 cm. Beyond their apertural regions, tunnel diameters increase very gradually towards, and reach a maximum at their distal ends, reflecting their acutely clavate morphology. Maximum diameters of exposed borings vary with the widths of their teredinid occupants and locally exceed 35 mm.

Distal termini of borings are typically rounded (hemispherical) and smooth; bioglyphs have not been observed. Cross-sectional outlines of borings are essentially circular along the axis of the tunnels. Slight deviations from circularity are observed locally where axes are contorted, and only in the narrowest, most proximal parts of borings (i.e., at the aperture) do cross sections exhibit figure-eight configurations that accommodate the shipworm’s paired siphons (Fig 5E)

The walls of *L*. *abatanica* borings are sharp and commonly truncate the tests of relatively thin-shelled foraminifera (Fig 3A). Borings are lined with calcite tubes that extend along the entire tunnel axis (e.g., Fig 5D). Linings range from <0.5 to 2.5 mm in thickness and typically comprise multiple layers that reflect distinct calcite secretion episodes (Fig 3A and 3B). Interiors and exteriors of linings are generally smooth, although both exhibit subtle transverse annulations spaced 3 to 15 mm apart (Fig 5D) that presumably reflect episodic growth of and boring by the tracemakers. In the most proximal parts of the borings, calcite tubes include a short partition or septum that separate the siphonal apertures and, in some specimens, may be thickened and extend slightly above the substrate surface (Fig 5E). Other accessory features commonly associated with modern and fossil teredinid tube linings—e.g., terminal or retrusive caps, and concamerations [1, 4, 15]—have not been observed.

Interiors of thinner linings locally exhibit, in isolation or in clusters, tiny low-relief (≤1 mm) bumps (Fig 5F), reflecting precipitation of linings over larger sand-sized skeletal allochems that intruded from the host limestone. Calcite tube exteriors locally exhibit dimples or pits of comparable size (Fig 5G) that reflect the same process. Both the interior bumps and exterior dimples can be considered xenoglyphs.

## Discussion

### Comparison with ichnotaxa attributed to bivalve bioeroders

Clavate ichnofossils attributed to boring bivalves are recognized in a variety of firm to hard substrates (e.g., [3, 16–17]. Those found in semiconsolidated sediment substrates (firmgrounds), carbonate hardgrounds, rockgrounds, shells, and bones are referred to the ichnogenus *Gastrochaenolites* or, less commonly, to the ichnogenus *Phrixichnus*. In contrast, clavate borings in xylic substrates (woodgrounds) are referred to the ichnogenus *Teredolites*. The character of these ichnogenera and associated ichnospecies are summarized in Table 1.

**Table 1:** Characteristics of generally clavate ichnotaxa in firm, hard (lithic), or xylic substrates and commonly attributed to boring bivalves.

Ichnotaxa	Diagnosis/description
***Gastrochaenolites*** [18]	Clavate borings in lithic substrates; apertural region narrower than main chamber and may be circular, oval, or dumbbell-shaped; aperture may be separated from main chamber by a neck region that in some cases is widely flared; main chambers vary from subspherical to elongate, have parabolic to rounded bases and circular to ovate cross sections, modified in some forms by a longitudinal ridge or grooves resulting in almond or heart-shaped cross sections; axes of borings may be straight, curved or irregular; boring may be lined, smooth walled or exhibit bioglyphs or xenoglyphs; typical diameters and lengths, 2 to 45 mm and 3 to 100 mm, respectively.
*G*. *lapidicus* [3]	Smooth-walled, clavate or elongate ovate borings; cross sections are circular throughout except for apertural region (ovate); base bluntly paraboloid in longitudinal section; widest diameters near center of main chamber; may be lined
*G*. *ampullatus* [3]	Smooth walled borings; main chambers are subspherical to elongate with circular cross sections; neck is flared and thickly lined to produce two diverging tubes leading to twin apertures; maximum diameters near center of main chamber
*G*. *cluniformis* [3]	Smooth-walled borings with a principal ridge and weakly developed ridge on diametrically opposed sides of main chamber; base is round to bilobate; neck and aperture are circular to ovate in cross section
*G*. *dijugus* [3]	Smooth-walled borings in which neck is constricted to form figure-eight cross section; neck region may have linings that extend above substrate surface as chimneys
*G*. *ornatus* [3]	Unlined borings circular in cross section throughout; deepest portion characterized by circular or spiral bioglyphs or serrated grooves
*G*. *torpedo* [3]	Elongate, smooth-walled boring with acutely parabolic base; widest point is close to the mid line; neck region is markedly compressed but aperture is oval or figure-eight shaped; commonly lined, with lining extending above substrate as chimneys
*G*. *turbinatus* [3]	Smooth-walled, acutely conical borings with evenly tapered body and neck; circular cross sections throughout; widest point near the rounded base; no known linings
*G*. *orbicularis* [3]	Smooth walled borings with orbicular main chamber and short to elongate neck; circular in cross section throughout; may exhibit thin lining
*G*. *cor* [19]	Smooth borings with somewhat discoid main chamber having a heart-shaped cross-section that is emphasized by a weak furrow running along both edges; furrow fades out in neck region; neck short and aperture round to oval, rarely reniform
*G*. *anauchen* [20]	Straight borings with smooth sides and circular cross section throughout length; expands gradually below the aperture, with greatest diameters about three-fourths of the depth; bases are rounded; no distinguishable neck
*G*. *oelandicus* [21]	Borings with irregular vase-like shape with roughly circular cross-section through the length of the structure; aperture is narrow; proximal (upper) portion is long and neck-like; diameter expands downward from the neck and then contracts again, resulting in an irregularly ovoidal form; base is irregular, typically tapering but may be flat
*G*. *pickerilli* [22]	Elongate, smooth borings of circular cross-section with calcareous lining; lateral, calcareous meniscate structures parallel one side of the borehole; train of menisci up to 2 cm long
*G*. *hospitium* [23] (= *G*. *vivus* [24])	Elongated, subcylindrical bivalve boring in a host coral, with two or more hemispherical bottoms (false floors) stacked at basal end
***Phrixichnus phrix*** [16]	Clavate boring with wall ornament consisting of arcuate or concentric grooves in two gently concave or flat areas that meet along one edge of the boring
***Teredolites*** [18]	Clavate borings in wood substrates; acutely turbinate, evenly tapered from aperture to base of main chamber; cross-section generally circular throughout; short to elongate, straight, sinuous, or contorted axes; borings may be lined
*T*. *clavatus* [18]	Clavate borings predominately perpendicular to wood substrate grain; Length/width ratios typically <5
*T*. *longissimus* [3] (= *Apectoichnus longissimus* [25])	Clavate borings predominantly parallel to wood substrate grain; commonly exhibit sinuous and contorted axes and calcite linings; Length/width ratios commonly >5;
*Borings of L*. *abatanica*	Highly elongate (L/W ratios >10), straight to highly sinuous borings with hemispherical base, circular transverse cross-sections (except for dumbbell shapes near aperture) and continuous thin to thick calcite linings; diameters increase gradually along axis, reaching a maximum near distal terminus

Morphological criteria used to distinguish ichnospecies of *Gastrochaenolites* and similar borings in lithic substrates (i.e., *Phrixichnus*), include (1) longitudinal profile (presence/absence and character of apertural necks, axial position of maximum tunnel width); (2) transverse cross-sectional profiles (e.g., circular, ovate, or dumbbell-shaped) and axial variations thereof; (3) shape of the basal terminus (e.g., parabolic, hemispherical, tapered, or flat); (4) presence and character of tunnel-wall bioglyphs or precipitated calcite linings; and/or (5) presence or absence of features indicative of retrusive vertical movement (i.e., false floors produced by equilibrium movement in response to growth of a living host—e.g., coral) or lateral migration through the substrate.

The borings of *L*. *abatanica* clearly differ from several of the ichnotaxa listed in Table 1. They lack the wall ornamentation (e.g., bioglyphs) diagnostic of *G*. *ornatus*, *G*. *cluniformis*, and *Phrixichnus phrix*, and lack features indicative of retrusive or lateral tube migration seen in *G*. *pickerilli* and *G*. *hospitium*, respectively. The Abatan borings do share some characteristics of remaining *Gastrochaenolites* ichnospecies, including smooth walls, predominance of circular transverse cross-sections, and presence of calcite linings observed in most ichnotaxa; the thickly lined, dumbbell-shaped apertures seen in *G*. *lapidicus* and *G*. *dijugus*; and the evenly tapered body and neck and rounded base characteristic of *G*. *turbinatus*. Nevertheless, *L abatanica* borings differ from previously defined *Gastrochaenolites* ichnospecies, particularly with respect to longitudinal profile; they are unique in that their tunnels are relatively long (L/W ratios >10), the expansion of tunnel diameter from aperture to tunnel base is very gradual yet continuous, and tunnel axes are commonly highly sinuous or contorted. The latter trait reflects the equant valves of the teredinids, which facilitate changes in direction of tunneling. With respect to length and axial distortion, *L*. *abatanica* borings are morphologically more allied with *Teredolites longissimus*, which as currently defined is limited to xylic substrates.

*Teredolites* ichnospecies are distinguished on the basis of axial orientation relative to substrate surfaces and L/W ratios (Table 1). Borings in wood assigned to *T*. *clavatus* are generally oriented perpendicular to substrate grain, have relatively straight axes, and are typically short (L/W ratios <5). In contrast, borings assigned to *T*. *longissimus*, like those produced by *L*. *abatanica*, are typically elongate (L/W ratios commonly well in excess of 5), exhibit sinuous to highly contorted axes, and are commonly partly or wholly lined by a calcite tube. These differences are linked to the general shell morphologies and feeding habits of their respective producers [4]. *T*. *clavatus* is normally associated with pholadid bivalves, which are filter-feeders; they are incapable of digesting cellulose [26–27] and thus tunnel mainly to construct a safe harbor from which to filter-feed. Moreover, pholadids are typically characterized by elongate shells that restrict mobility within their tunnels and limit axial distortion of borings [28]. In contrast, *T*. *longissimus* is associated with teredinids, the majority of which, although capable of facultative filter-feeding [1, 27–29], are obligate wood-eaters; they digest and metabolize cellulose and thus continue to bore as long as substrate space is available. More importantly, their equidimensional valves permit free anterior rotation and distortion of their tunnel axes [28]. Notably, *T*. *longissimus* in modern and fossil substrates typically contain a tube lining that may include internal accessory features (e.g., concamerations, anterior and retrusive caps) that reflect the ability of teredinids to seal one or both ends of their tunnels [1, 4, 15].

In general practice, the application of ichnotaxonomic names to modern biogenic structures should be avoided (but see [30] re lithic substrates). However, it is important to consider how fossil borings comparable to those produced by *L*. *abatanica* would be classified in the ichnotaxonomic sense. Given their presence in lithic substrates and overall clavate morphology, such borings could be assigned to *Gastrochaenolites*. Considering their unique features (e.g., axial length and tortuosity), however, these structures would require establishment of a new ichnospecies, an option beyond the scope of the current contribution. Alternatively, such borings may be regarded as a different ichnogenus altogether. Donovan [25] recently argued that substrate type is a poor ichnotaxobase and highlighted what he deems to be significant morphological differences between *T*. *clavatus* and *T*. *longissimus*; i.e., the much-reduced rate of tunnel-width expansion and axial distortion manifest in *T*. *longissimus*. Thus, Donovan proposed that the latter be reassigned as the type ichnospecies of a new ichnogenus, *Apectoichnus*. If Donovan’s ichnotaxonomic arguments are accepted, the *L*. *abatanica* borings could be considered as incipient forms of *Apectoichnus*.

*Lithoredo* borings suggest a thought experiment that contributes to the debate on whether substrate is an appropriate ichnotaxobase [25, 30–31]. At some point in the evolutionary history of the *Lithoredo* lineage, individuals made the transition from boring in wood to boring in rock. The morphology of the valves then must still have been functional for boring in wood. The ability to bore in rock is an exaptation—the same behavior of the animal that allowed it bore in wood, also allowed it to bore in rock. Consider a piece of wood trapped in a crevice of the rock. In principle, an animal boring through the rock could continue boring through the wood. Would the trace fossil then change partway through from *Gastrochaenolites* to *Teredolites*? Or, should the name *Apectoichnus* be applied based on morphology, disregarding substrate? The point in using substrate as an ichnotaxobase is that substrate implies a different behavior by the trace producer even if the traces are morphologically similar [30, 32]. At the time of the cladogenetic event that led to *Lithoredo abatanica*, there likely was not a difference in boring behavior. We do not know, however, if boring behavior in *Lithoredo* has remained the same, or whether *Lithoredo* can still bore in wood. The valve morphology has changed substantially from the plesiomorphic form, but this could just make the same boring behavior more effective in rock. But boring behavior could also be modified, or behaviors added, perhaps acid secretions to soften the rock, as in other bivalves that bore in hard substrates [33–34].

Bertling et al. [30] note that the divisions among “soft, firm and hard substrates are real but not sufficiently distinct; a few exceptional boring organisms may attack stiff mud as well as lithic substrates”. They advocate keeping nomenclature of trace fossils in lithic, xylic and soft substrates separate “regardless of morphologic similarity (not identity)” and conclude that “It would usually be a mistake, however, to name a new ichnotaxon based solely on a difference in substrate.” Are *Lithoredo* borings another rare exception to the rule, or do they demonstrate that substrate is not an appropriate ichnotaxobase? We cannot answer that question here, but suggest that careful consideration of the evolutionary origins of the behaviors of trace makers may provide guidance.

### Teredinids, substrates, and nutrition

As previously noted, most shipworms are well adapted for life within woody substrates: they possess denticulated valves, which, in combination with a large posterior abductor muscle, facilitate drilling in wood; a wood storing organ (caecum), which is also the primary site of wood digestion; and, intracellular cellulolytic bacterial symbionts, harbored in their gills, that produce enzymes that further aid in wood digestion [1, 35–37].

The shipworm *Kuphus polythalamius*, found in the modern Indo-West Pacific region [38], represents an exceptional form that is not limited to woody substrates but, at least in later stages of development, is adapted to burrowing in soft marginal marine and marine sediments, including carbonaceous muds 1, 11, 38]. These sediment dwellers, some of which may reach enormous sizes (up to 1.6 m long and 7 cm in diameter), have a diminished capacity for drilling into and digesting wood; their valves lack sharp sculptured teeth, posterior abductor muscles are comparatively poorly developed, and digestive systems lacks a cecum [38]. Rather, *K*. *polythalamius* harbors sulfur-oxidizing chemoautotrophic (thioautotrophic) bacteria, indicating that these sediment-dwelling shipworms may be chemoautrophic; i.e., they exploit H_2_S as a source of nutritional energy [11, 39].

While it is evident that *L*. *abatanica* is lithophagous—i.e., it ingests the limestone substrate into which it bores—the feeding mechanism of this newly recognized shipworm is not known. *Lithoredo abatanica* may feed on particulate organic matter (planktonic algae, bacteria or terrestrial plant detritus) in river water, harvest microbes (e.g., green algae, cyanobacteria) embedded in substrate pores, and/or, like *K*. *polythalamius*, exploit intracellular symbionts [12]. How these shipworms derive their nutritional energy is the subject of ongoing study. Whatever their means of sustenance, the current study documents another exception to obligate xylotrophy/xylotrepesis in Teredinidae, demonstrating for the first time that shipworms, like their pholadid relatives, are capable of boring into lithic substrates.

### Teredinids & salinity

Body fossils and biogenic structures (*Teredolites*) of shipworms thus far found in the stratigraphic record are limited to woody substrates, either composite xylic substrates now manifest in coals or lignites [5, 40] or in isolated lignitized wood clasts (log-grounds) found in variable concentrations within clastic or carbonate sediments (e.g., [4, 6–9, 15, 41]. With one rare and questionable exception [40], occurrences of fossil shipworm-bored wood are limited to marginal marine (estuarine) or marine (mainly shelf) sedimentary successions. Hence, occurrences of *Teredolites longissimus*, as well as *T*. *clavatus* associated with boring pholadids, traditionally have been employed in depositional facies analysis as criteria for recognizing marine influence. However, the current study, along with previous investigations of modern bivalve ecology elsewhere, demonstrates that shipworms and their ichnofossils may not be strict indicators of ancient brackish or marine conditions.

The bioerosion by *L*. *abatanica* documented here clearly occurs in freshwater reaches of the Abatan River, well upstream from its brackish estuarine mouth. Moreover, many of the submerged logs on the river bed and submerged living tree roots along the river bank at this same locality are inhabited by the wood-boring shipworm *Nausitora* sp. and are densely riddled with its *T*. *longissimus*-like borings. Notably, *Nausitora* has been previously recognized in similar freshwater fluvial settings at various localities worldwide [1], and another shipworm—*Psiloteredo healdi*—is a freshwater specialist found in rivers and lakes in Central and South America; e.g., the Surinam River (Suriname), Comprido and Escuro rivers (Brazil), Lake Maracaibo (Venezuela) (e.g., [1, 42–43].

The modern freshwater-adapted shipworms that have been documented to date occur in settings that are connected to estuarine marine water bodies. In a recent study similar to our own, Bolotov et al. [44] documented an unusual freshwater occurrence of a bioeroding pholadid bivalve—*Lignopholas fluminalis*—that produces borings analogous to *Gastrochaenolites anauchen* in submerged siltstone outcrops beneath freshwaters of the Kaladan River (Myanmar) ~71 km from its estuarine mouth. These authors proposed that recent tectonic uplift, gradual sea-level fall, and the consequent transition from estuarine to freshwater conditions in their study area played a major role in the environmental transition of *L*. *fluminalis* and associated macrofauna. It is conceivable that the evolutionary adaptation of shipworms, including *L*. *abatanica*, to freshwater systems similarly may have been driven by Quaternary or earlier sea-level dynamics.

At present, it is not yet clear when extant shipworm species made the transition to freshwater systems or whether similar adaptations have occurred at times in the geologic past. The same uncertainty exists for the freshwater rock-boring pholadids such as those documented by Bolotov et al. [44]. Hence, given the potential for shipworms to adapt to freshwater aqueous settings, caution is called for in the interpretation of ancient depositional environments based solely on occurrences of *Teredolites* in wood substrates or *Gastrochaenolites* in lithic substrates.

### Shipworms as ecosystem engineers & biogeomorphic agents

Considerable attention has been given to macrobioerosion of lithic substrates, particularly carbonate rocks, in modern and ancient marine settings. Such studies have focused on bioeroding organisms, the biogenic structures they produce (e.g., [2, 45–49], and their roles as ecosystem engineers and geomorphic agents (e.g., [50–53]. In contrast, the potential for macrobioerosion of lithic substrates in freshwater aquatic systems has only recently been recognized (e.g., [44, 54]). Observations made in the current study, demonstrate that shipworms may have a broader range of impacts than previously recognized. Historically, shipworms have been recognized as the primary bioeroders of lignocellulosic materials in shallow coastal marine environments, including mangrove roots, seagrass rhizomes and terrestrial woods, as well as coastal constructions (piers, docks, jetties), fishing equipment and wooden sailing vessels. Observations described herein, indicate that shipworms also may serve as important ecosystem engineers and geomorphic agents in freshwater settings. The bioerosion by *Lithoredo abatanica* has enhanced the habitat complexity of the Abatan River bed. Notably, abandoned shipworm tunnels here host a variety of nestling organisms, including crabs, shrimp, limpets, gastropods, clams, and polychaetes [12]. Moreover, dense emplacement of borings in these limestone substrates by these lithophagous shipworms likely have a significant impact on rates of physical and chemical erosion of the stream channel, particularly during times of higher stream discharge. Indeed, the geomorphic impacts that these freshwater bioeroders have on landscape development may be comparable to those of bioeroders in more thoroughly studied marine coastal settings.

## Summary

Moderately indurated limestones exposed in the banks and bed of a freshwater stretch of the Abatan River, southwestern Bohol, Philippines, serve as substrates for *Lithoredo abatanica*, a recently recognized taxon of Teredinidae. This unique lithophagous shipworm is responsible for extensive macrobioerosion of the limestone substrate, producing networks of elongate, acutely clavate, axially contorted, calcite-lined borings. Given their strong resemblance to borings produced by teredinids in woody substrates, but their production in a lithic substrate, *L*. *abatanica* borings can be regarded as incipient forms of *Gastrochaenolites* sp. or *Apectoichnus sp*. pending the outcome of debate regarding the efficacy of substrate type as an ichnotaxobase. The discovery of shipworm macrobioerosion of lithic substrates in a freshwater fluvial setting has implications for interpreting ancient depositional conditions based on occurrences of shipworm body fossil and ichnofossils. In addition, these findings demonstrate that the roles of macrobioeroders as ecosystems engineers and biogeomorphic agents in freshwater aquatic settings may be on par with those documented in more thoroughly studied marine and marginal marine systems.
